# Is Hogarth’s ‘Line of Beauty’ really the most beautiful? An empirical answer after more than 250 years

**DOI:** 10.1177/20416695221087738

**Published:** 2022-03-31

**Authors:** Ronald Hübner, Emily Ufken

**Affiliations:** 26567 University of Konstanz, Konstanz, Baden-Württemberg, Germany; 9377 Phillips-University of Marburg, Marburg, Hessen, Germany

**Keywords:** line of beauty, curvature, William Hogarth, line aesthetics, serpentine

## Abstract

Since the Renaissance, different line types have been distinguished by artists and art theorists. However, it took another hundreds of years until the British artist William Hogarth attributed different degrees of beauty to them. Particularly, in his book “The Analysis of Beauty” (1753) he depicted seven waving lines, declared line number 4 as the most beautiful, and called it the “line of beauty”. Until today, the line of beauty has a persistently strong influence in many areas such as landscape art and design, calligraphy, furniture design, architecture, dance, etc. It is astonishing that Hogarth's assumptions have never been empirically tested. Therefore, we asked participants to rate Hogarth's lines by their beauty. As a result, line number 4 was indeed the most preferred, although number 5 was judged similarly. An analysis revealed that curvature was nonlinearly related to beauty and explains more than 90% of the variance in the mean aesthetic judgments.

Although line drawings already occurred in prehistoric art (Kennedy, 1975), they played little role in the first following periods. Only from the renaissance, contour lines were defined and discussed in the writings of that time (Jacobs, 1988). The development can be traced back to the German painter, printmaker, and theorist Albrecht Dürer (1471–1528), who, in his book “Instructions for Measuring with Compass and Ruler” (Dürer, 1525), distinguished between straight lines (“gerade Lini”), circle lines (“zirkel Lini”), and serpentine lines (“chlangen Lini”). However, Dürer did not attribute any aesthetic properties to the individual line types. This changed to some extent with the Lombard painter and theorist Giovanni Paolo Lomazzo (1538–1592), who recommended in his writings (Lomazzo, 1584/1844) to make figures to seem more dynamic by merging a pyramid and an S-form into a serpentine shape (Rosenberg, 2007). He called this shape “figura serpentinata” and attributed the naming to the Italian sculptor, painter, and architect Michelangelo Buonarroti (1474–1564).

An important step towards a line aesthetics was the paradigm shift in art from mimesis (nature imitation) to a reception-oriented theory of art in the 18th century, which emphasized the relationship between the artwork and the viewer, and which increased the interest in the formal properties of an artwork responsible for effects on art appreciation beyond object representation (Rosenberg, 2007). In the course of this shift, the British artist, printmaker, and theorist William Hogarth (1697–1764) published his book “The Analysis of Beauty” (Hogarth, 1753), in which he distinguished straight, curved, waving, and serpentine lines. Whereas the first three types were pure two-dimensional, he defined serpentine lines as winding and, if drawn, requiring a three-dimensional interpretation. Most importantly, however, Hogarth not only distinguished between these line types, but also normatively attributed different degrees of beauty to them. He called waving lines “lines of beauty” and serpentine-lines “lines of grace”. In a pioneering approach, he assessed the beauty and grace of these lines by observing their variation, use, and aesthetic evaluation by others. Accordingly, the founder of empirical aesthetics, the German experimental psychologist, philosopher, and physicist Gustav Theodor Fechner (1801–1887) considered Hogarth's method as an early example of “working from below” (Fechner, 1876). Most importantly, however, in view of his fundamental achievements, Hogarth can be regarded as the initiator of line aesthetics (Rosenberg, 2007).

Interestingly, his ideas received little credit from his British contemporaries (Gombrich, 1951). However, they were well received in other countries, especially in Germany. By the order of the Prussian king Frederick the Great and under Hogarth's supervision, Christlob Mylius (1722–1754) soon translated “The Analysis of Beauty” into German. The translation was already released in 1754. The German writer, philosopher, and dramatist Gotthold Ephraim Lessing (1729–1781) was so enthusiastic about Hogarth's book that he wrote an introduction to the second edition (Heier, 1977). An Italian translation appeared in 1761, followed by a French version in 1805 (Dobson, 1907), and a Russian translation in 1958.

From all of Hogarth's ideas, his line of beauty had the greatest impact. For instance, already shortly after publication of “The Analysis of Beauty”, it strongly influenced the aesthetic concepts of the German poets and playwriters Friedrich Schiller (1759–1805) and Johann Wolfgang von Goethe (1749–1832) (Heier, 1977). Furthermore, until today the line of beauty has been considered as the ideal in such diverse fields as landscaping and river design (Myers, 2004; Podolak & Kondolf, 2016), calligraphy (Howard, 1985), furniture (Vallance, 1913), hairdressing (Saviello, 2017), poster (propaganda) composition (Erwin, 2001), or dance (Lindberg, 1980; Richardson, 2002). Recently, Hogarth's idea even found its way into a novel called “Line of Beauty” (Hollinghurst, 2005).

Given Hogarth's innovative ideas and his enduring great influence in various fields of aesthetics, art, and design, it is surprising that his line of beauty is largely ignored in research on empirical aesthetics. This is the more astonishing as the *curvature effect* has intensively been investigated, i.e., the hypothesis that curved shapes are preferred over angular ones (e.g., Bar & Neta, 2006; Bertamini et al., 2016; Carbon, 2010; Corradi et al., 2019; Gómez-Puerto et al., 2017; Palumbo & Bertamini, 2016). Admittedly, compared to the curvature effect, the line of beauty is more specific. However, it is also commonly assumed that waving lines are more beautiful than straight lines and even more beautiful than curved lines.

It should be noted that in the cited literature on Hogarth, waving lines are often generally called “line of beauty” irrespective of their specific features. However, Hogarth was quite clear about which features a waving line must have to be called line of beauty. In reference to one of his illustrations (see Figure 1) he wrote:

**Figure 1.**
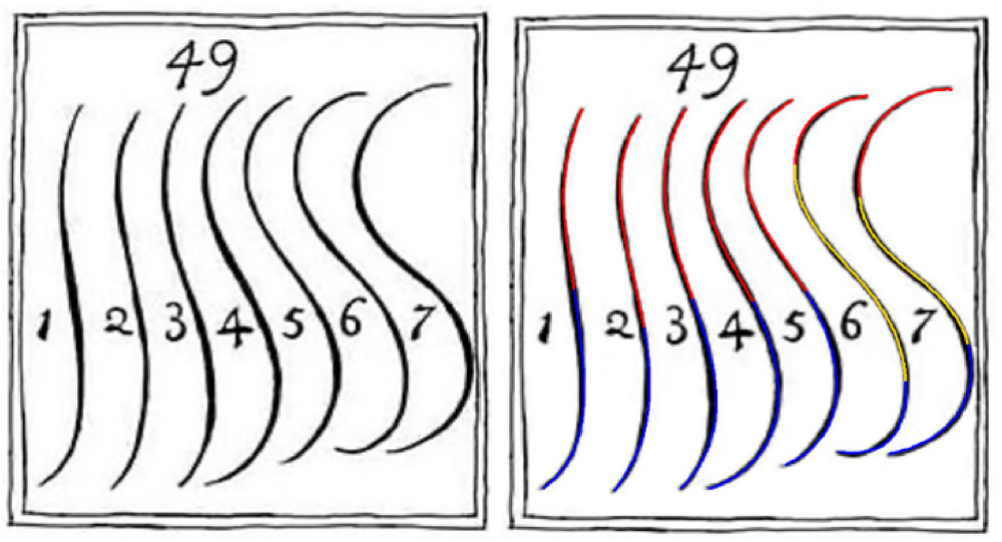
The figure with the waving lines from the book “The Analysis of Beauty” (Hogarth, 1753). Left panel: Copy of the original figure (Downloaded from: https://commons.wikimedia.org/wiki/File:Serpentine_lines_from_William_Hogarth%27s_The_Analysis_of_Beauty.jpg). Right panel: The lines superimposed with fitted composite Bézier curves. The red, blue, and yellow parts indicate the two or three different cubic Bézier component curves, respectively.

“Though all sorts of waving-lines are ornamental, when properly applied; yet, strictly speaking, there is but one precise line, properly to be called the line of beauty, which in the scale of them, Figure 1, plate 16, is number 4: the lines 5, 6, 7, by their bulging too much in their curvature, becoming gross and clumsy; and, on the contrary, 3, 2, l, as they straighten, becoming mean and poor; …” (p. 49).

This specific historic statement about the line of beauty has led us to ask: Is line number 4 really the most beautiful? And if so, what properties are responsible? The aim of the present study was, after more than 250 years, to answer these questions.

For answering the first question, we asked participants to scroll through the seven lines in Figure 1 and select the one they found most beautiful. Additionally, in a subsequent task the participants had to rate each curve how beautiful they find it. For answering the second question, we fitted composite lines, composed of cubic Bézier curves, to the original lines. For lines 1 to 5 two cubic Bézier curves were sufficient for a good fit, whereas three curves were needed for lines 6 and 7. The fitted lines allowed not only a parameterization of Hogarth's waving lines but also made it easy to compute their properties such as curvature.

## Method

### Participants

Seventy participants (mean age 23.6 years, SD = 4, 18 male), who were students at the University of Konstanz, at the Phillips University of Marburg, or not associated with the universities, participated in the study. For participation, they could win one out of 6 Amazon vouchers worth €10. The experiment was conducted in accordance with the ethical guidelines of the University of Konstanz and the Declaration of Helsinki (1964) and its later amendments. Participants were informed of their right to quit the study at any time without reprisal, and their informed consent was obtained by check-marking a box before the actual experiment started.

### Stimuli

**Figure 2.**
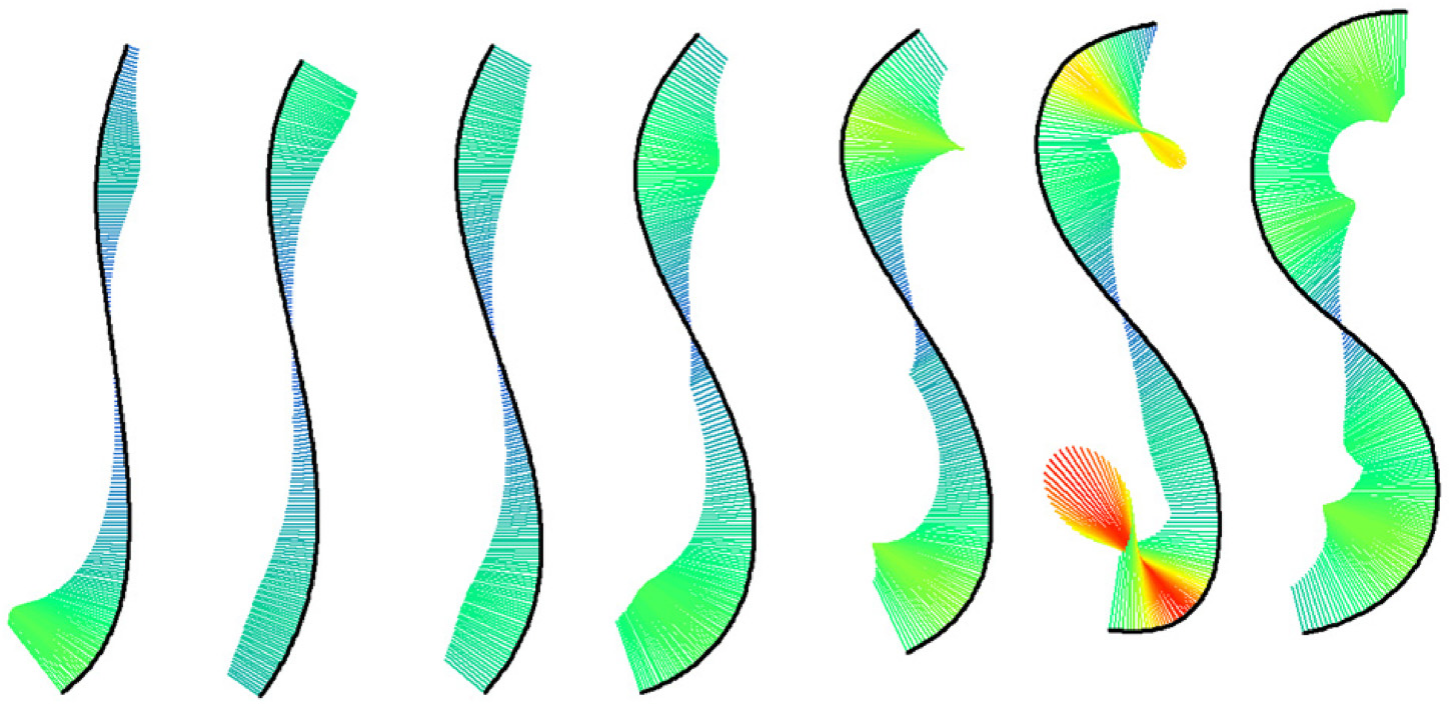
Lines fitted to the seven lines in Figure 1 and their curvature. Curvature at points along the lines is indicated by the length (scaled for good visibility) and color (redundant coding) of the lines orthogonal to the tangent at that point.

As stimuli, we served digitized versions of the seven Hogarth lines (Figure 1). As already implied by Hogarth's statement cited in the Introduction, it is likely that differences in preference are mainly due to differences in curvature. For examining this relation, we used the R Package “knotR” (Hankin, 2017) and fitted composite lines consisting of several cubic Bézier curves to the Hogarth lines. A cubic Bézier curve is determined by four control points, each defined by its *x* and y coordinate. The first and last control points are the starting and end point of the curve, respectively, whereas the other two control points determine the curvature of the curve. In composite lines, the endpoint of the first Bézier curve is the starting point of the next one and so on. To obtain smooth transitions between the component curves, our fit procedure minimized the deviation of the composite line from the original one under the constraint that the components must have the same slope and curvature at the points where they join. Whereas lines 1 to 5 could well be fitted by two connected cubic Bézier curves, three curves were necessary for lines 6 and 7 (see Figure 1). Given a Bézier curve, the curvature of each point along the line can easily be computed. The curvatures for the seven Hogarth lines are shown in Figure 2.

By convention, the upper arc of an S-shape is convex and the corresponding curvatures positive, while the lower arc is concave and curvature negative. As the values of curvature are rather small, we transformed them to the *radius of curvature*, a measure defined by 1/curvature (Burchhard et al., 1994). We then computed the mean absolute radius of curvature (RC) for the seven Hogarth lines, and obtained 108, 109, 98, 66, 59, 48, and 48 (in pixels), respectively. These measures were later used to relate curvature to our data.

### Procedure

The program for this online experiment was written in Javascript and ran in the browser used by the respective participant. The tasks for the present study were the first in a larger study. The subsequent tasks had different objectives and their results will be reported elsewhere. At the beginning of this study, the participants were asked for consent and demographic information. Then they had to perform two tasks, each introduced by an instruction. In a choice (selection) task the participants saw a single curve but could scroll back and forth through the ordered Hogarth lines by pressing the left or right arrow key on the keyboard, respectively. Scrolling did not reach an end. Rather, the lines appeared in the order … 1 2 3 4 5 6 7 6 5 4 3 2 1 … or the other way round, depending on the scrolling direction. The starting line was randomized, and the participants were instructed to first scroll through all different lines and then to display the line they find most beautiful. The choice was finalized by hitting a NEXT button with the mouse.

Subsequently, there was a rating task in which the participants saw the individual seven lines in random order and had to rate their beauty on a visual analog scale, internally ranging from 1 to 100.

## Results

The correlation between the number of the chosen line and the number of the line with the maximum rating was.387, *t*(68) = 3.46, *p* < .001. The relatively low correlation was mainly due to nine participants. One of them selected line number 1, and most of the others line number 7. In the beauty rating they then responded rather inconsistently. Without these nine participants the correlation increases to.743, *t*(59) = 8.52, *p* < .001. However, because the data of the nine participants did not affect the other results, they were not excluded from further analyzes.

**Figure 3.**
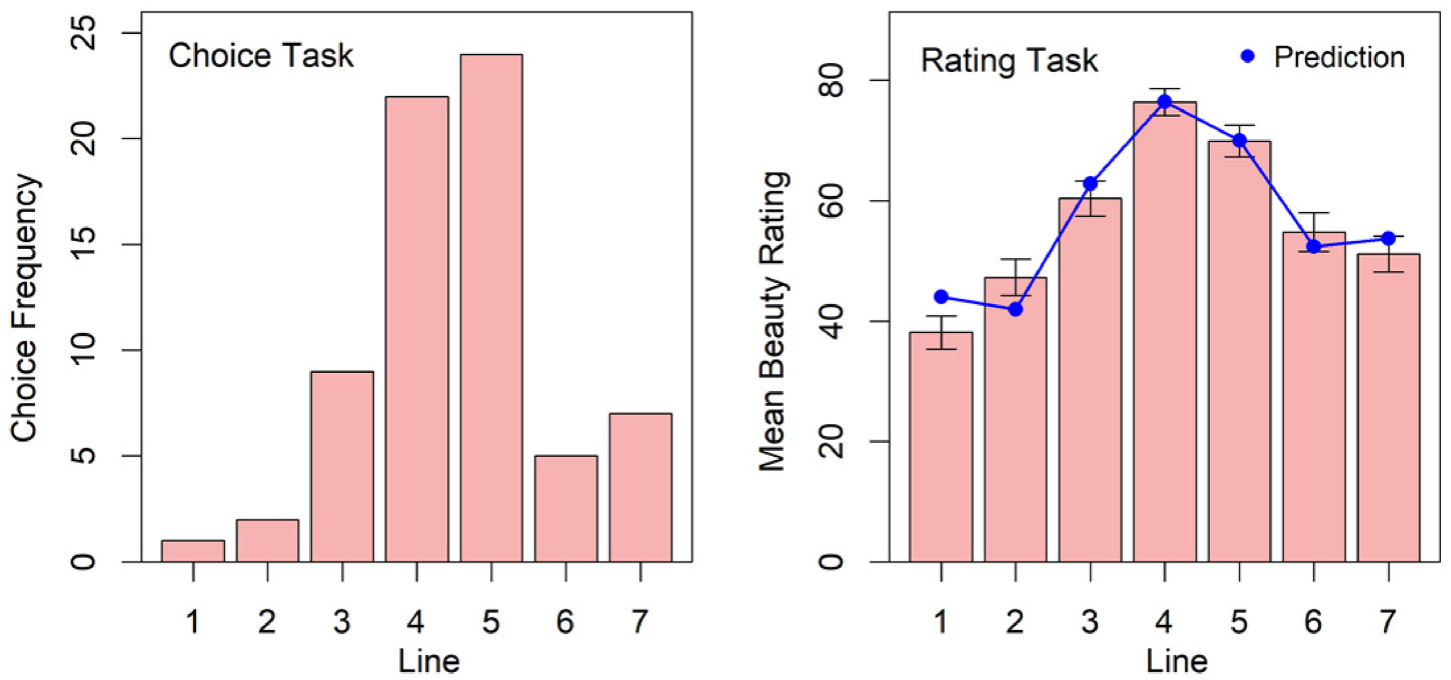
Results in the choice task (left panel) and rating task (right panel) with the seven Hogarth lines (Figure 1). The error bars in the right panel represent the standard errors. The blue dots represent the predictions by our multiple-regression model (see Text for details).

The choice frequency for the individual curves in the choice task are shown in the left panel of Figure 3. As can be seen, lines number 4 and number 5 were chosen most often, where number 5 was slightly more frequently chosen than number 4.

The ratings show a similar result. An ANOVA (analysis of variance) revealed significant differences between the ratings, *F*(3.44, 237.6) = 27.3, *p* < .001, GES = 0.209. Line number 4 was rated as most beautiful, closely followed by number 5. Pairwise comparisons of line number 4 with all other lines revealed a significant difference (Bonferroni adjusted) with all lines except number 5, *t*(69) = 2.36, *p*_adj_ = .125.

**Figure 4.**
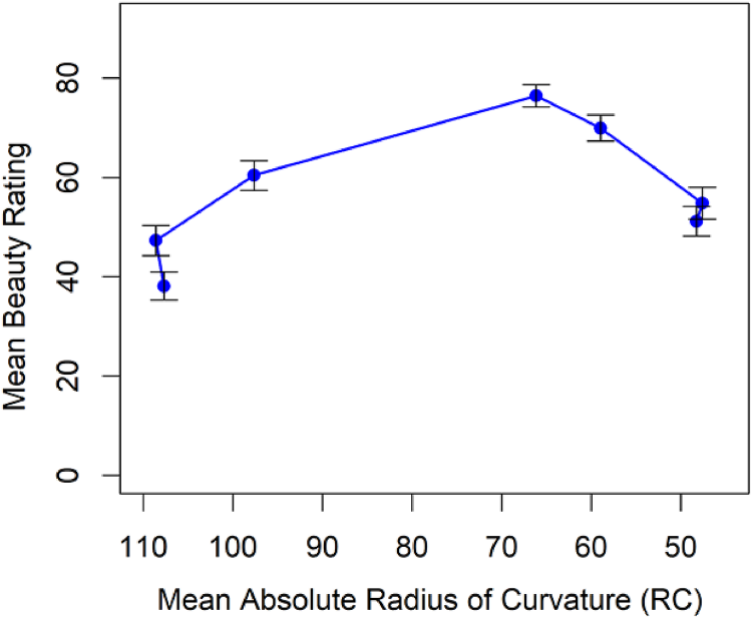
Mean beauty ratings of the seven Hogarth lines (Figure 1) as function of the mean absolute radius-of-curvature (RC).

Because beauty increased with curvature up to some level and then decreased (see Figure 4), the two variables were obviously related non-linearly. Accordingly, we computed a multiple linear regression, where the dependent variable *mean beauty* (*B*) was predicted by a quadratic polynomial with our radius-of-curvature measure RC as independent variable. Specifically, the equation is:

(1) $$B=a_{0}+a_{1}RC+a_{2}RC^{2}.$$

As result, equation (1) explains more than 90% of the variance, R^2^ = 0.926, *F*(2, 4) = 25.0, *p* < .01. Table 1 shows the detailed result. In the right panel of Figure 3 the connected (blue) dots represent the predicted mean beauty.

**Table 1.** Result of the Multiple Regression Analysis. The Coefficients are Defined in Equation (1). *p < .05, **p < .01.

Coefficient	Estimate	Std. error	*t*	*p*
$a_{0}$	−109	30.3	−3.61	.0226*
$a_{1}$	4.98	0.850	5.86	.004**
$a_{2}$	−0.033	0.005	−6.15	.004**

## Discussion

Our results are largely in line with William Hogarth's assertion that his line number 4 is the most beautiful of his seven waving lines (see Figure 1). More than 250 years after the lines were published, our participants rated line number 4 as most beautiful, although the rating of number 5 did not differ significantly. In the choice task, line number 5 was even chosen most frequently as most beautiful, followed by line number 4. The slightly different numerical results in the two tasks are presumably due to the different methods. In the selection task, there can be a contrast effect, because the participants scrolled trough ordered curves, while this was not possible in the rating task, where the presentation of the different curves was randomized. In any case, it seems justified to conclude that the lines number 4 and 5 are equivalent with respect to their beauty.

While our results are compatible with Hogarth's ideas, do they also show that line number 4 is the line of beauty? We think that this is not necessarily the case. Besides the fact that line number 5 was similarly rated, it should be noted that line number 4 was not only the central line but also presented, as in Hogarth's figure (Figure 1), in the context of a specific selection of other lines. Thus, the observed judgements were relative. The crucial question therefore is: What are the characteristics of the line of beauty? Hogarth's account, as mentioned in the Introduction, that less beautiful lines “bulge too much in their curvature, making them gross and clumsy”, or “as they straighten becoming mean and poor”, is not satisfactory from today's point of view. Aesthetic judgements should be related to objective measures. Our analysis revealed that mean curvature was non-linearly related to beauty and the most important feature for the corresponding judgements. Accordingly, a quadratic polynomial with mean absolute (radius of) curvature as independent variable explains more than 90% of the variance of the mean aesthetic ratings.

However, the successful account by curvature might be misleading. Because the seven lines also differ in other properties such as arc length, the length of the upper arc relative to the lower one, line thickness, etc., our measure of curvature might be confounded with some of these features. Thus, just by showing line preference with the Hogarth lines does not tell us exactly why the middle lines were preferred. Accordingly, our validation study was an important, but only first, step towards revealing the characteristics of the line of beauty. Therefore, it remains for future research to show exactly what properties make a waving line beautiful. One approach could be to specify one of Hogarth's ideas. Among his proposed six key principles of beauty are *uniformity* and *variety* both of which are fulfilled by the line of beauty. Unfortunately, other waving lines fulfill these rather general principles as well. However, they could possibly be specified in terms of curvature so that they uniquely describe the line of beauty. For instance, each line has its characteristic distribution of curvature along its arc, as can be seen in Figure 2. It is conceivable that a certain combination of attributes of this distribution leads to uniformity in variety and thereby causes the preference for the line of beauty.
